# Dimerization of Linear Butenes and Pentenes in an Acidic Zeolite (H‐MFI)

**DOI:** 10.1002/anie.202013671

**Published:** 2020-12-14

**Authors:** Fabian Berger, Joachim Sauer

**Affiliations:** ^1^ Institut für Chemie Humboldt-Universität zu Berlin Unter den Linden 6 10099 Berlin Germany

**Keywords:** alkanes, alkenes, Brønsted acid sites, dimerization, zeolites

## Abstract

Quantum chemical evidence is produced to show that dimerization of linear butenes and pentenes at zeolitic Brønsted sites in H‐MFI yields alkanes featuring cyclohexane rings rather than branched alkenes. The absence of any C=C double bond in the formed cyclic alkane explains the observations that oligomerization stops at the dimer. The calculated reaction enthalpies for the dimerization of 2‐pentene in the gas phase are −84 kJ mol^−1^ for branched alkenes, but −153 and −154 kJ mol^−1^ for alkyl‐cyclopentane and ‐hexane, respectively. Together with calculated adsorption enthalpies of the dimers, −111 and −127 kJ mol^−1^, respectively, this implies surface dimer formation enthalpies of −264 and −281 kJ mol^−1^, respectively, in close agreement with the experimental value of −285 kJ mol^−1^. In contrast, the predicted enthalpy for formation of branched alkoxides, −198 kJ mol^−1^, deviates by 87 kJ mol^−1^ from experiment. Calculated IR spectra for the Brønsted OH group show the observed conversion of the band at approximately 3000 cm^−1^ (hydrogen bond with alkene) to a less intense band at approximately 3450–3500 cm^−1^ (interaction with alkane).

## Graphical abstract

Dimerization of linear butenes and pentenes at zeolitic Brønsted acid sites yields, rather than branched alkenes, alkanes featuring a cyclohexane ring. The absence of any C=C double bond in the latter explains not only the observed change of the IR spectra, but also the observation that oligomerization stops at the dimer.

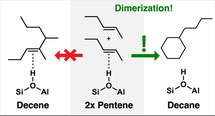

The catalytic conversion of alkenes on acidic zeolites is of high relevance for many industrial processes, see ref. [1] and references therein, but the atomistic details how alkenes interact with the Brønsted acidic sites (BAS) in zeolite pores are still poorly understood. The reason is that already at room temperature alkenes polymerize and possible surface species escape experimental characterization. Experimental studies (IR, calorimetry) of the oligomerization of butene^[2]^ and pentene^[3]^ could neither explain why oligomerization stops at the dimer nor provide a convincing assignment of observed spectra.

Here we show that dimerization of small alkenes does not form a *longer alkene* (which was assumed to bind as alkoxide to the surface)^[3]^ but an alkyl‐substituted *cycloalkane*, and that this explains all observations[^2^, ^3^, ^4^] in a natural way: (i) oligomerization stops at the dimer because there is no double bond left, (ii) the appearance of an IR band at about 3500 cm^−1^ typical of the interaction of an alkane with the zeolitic OH group, and (iii) the calorimetric measurement of the reaction enthalpy for the dimerization of pentene. Our conclusions are based on Density Functional Theory (DFT) for IR spectra, on chemically accurate Coupled Cluster theory with Single, Double and perturbative Triple substitutions (CCSD(T)) for gas phase dimerization energies, and CCSD(T)‐quality hybrid QM:QM calculations (QM—quantum mechanics) for adsorption energies.

Four different species may form on interaction of alkenes with the internal surface of acidic zeolites: Molecular adsorption may yield (i) a van der Waals complex with the pure silica wall or (ii) a π‐complex with the BAS,^[3]^ whereas protonation of alkenes may yield either (iii) alkoxides or (iv) carbenium ions,^[5]^ see also, e.g., ref. [6]. NMR evidence for alkoxides has been produced only after dehydration of alcohols,^[7]^ whereas on adsorption of alkenes, so far, spectroscopic signatures have been observed only for π‐complexes. Long ago Kondo et al.^[2]^ studied adsorption of 1‐butene on H‐MFI below room temperature (180 K) by FT‐IR spectroscopy and observed a broad band at 3100 cm^−1^ typical of a hydrogen‐bond between the OH group of a BAS and an alkene double bond, while at the same time the signal of the free BAS OH group at 3610 cm^−1^ was disappearing. The same observations have been made later for 1‐butene and *i*‐butene on H‐MFI at 298 K,^[4]^ and recently for the interaction of 1‐pentene with H‐MFI at 323 K.^[3]^

After increasing the temperature^[2]^ respective in the further course of the reaction,[^3^, ^4^] the groups observed the disappearance of the H‐bond band of the π‐complex and the reappearance of the signal for free OH groups, but only with about half intensity. This was interpreted as due to formation of an alkene dimer (D) which would only interact with half of the BAS (D/H‐Z) according to (M stands for the alkene monomer and H‐Z for the BAS):

(1a) 2M+2H-Z→2M/H-Z

(1b) 2M/HZ→D/H-Z+H-Z

At these temperatures double bond migration was observed,^[4]^ starting at 250 K,^[2]^ whereas skeletal isomerization occurred only after heating to 373 K.^[4]^ Much higher temperatures are required for alkene cracking (573 K)^[8]^ or alkene conversion via the hydrocarbon pool mechanism (623 K).^[9]^

The dimerization hypothesis left two questions open:

First, why did the H‐bond band disappear completely? The double bond of the branched alkene dimer should also form a π‐complex with the OH group of the BAS resulting in a H‐bond band with half intensity because two monomer complexes are transformed into one dimer complex.

Second, why did the polymerization stop at the dimer (which also features a double bond) and did not proceed to longer alkenes?

Kondo et al.^[2]^ explained this with steric hindrance. “The C=C bond of the produced dimer was found to be hindered to hydrogen‐bond with the OH groups of BAS […]. Therefore, protonation of the dimer would not occur, and further reactions such as oligomerization and polymerization would not proceed.” They observed a broad band at 3500 cm^−1^ which they assigned to hydrogen bonding with the alkyl chain of the dimer. This was based on the shift from 3609 to 3474 cm^−1^ observed on adsorption of heptane on H‐MFI by Spoto et al.^[10]^ Sanchez‐Sanchez, Lercher and co‐workers provided a different explanation.^[3]^ They suggested that the double bond in the dimer alkene disappeared (and with it the 3100 cm^−1^ band) because a surface alkoxide was formed. The band at 3450 cm^−1^ which they also saw appearing in the course of the reaction they considered weak and ascribed it to a “minority of molecules that interact in that way”, and they assumed that it is “the low concentration of molecules in the pores” that “limits the reaction to dimerization …”.^[3]^

These authors^[3]^ made an important step ahead and combined their IR experiments with gravimetry and calorimetry. For the formation of the dimer at the BAS from *trans*‐2‐pentene in the gas phase, P, they measured a reaction enthalpy, Δ*H*_r_, of −285 kJ mol^−1^.

(2) 2P+2H-Z→D/H-Z+HZ

It can be decomposed into the enthalpy Δ*H*_d_ of dimerization in the gas phase,

(3) 2P→D

and the enthalpy Δ*H*_s_ for binding the dimer species onto the zeolite,

(4) D+H-Z→D/H-Z

where D/H‐Z stands for any surface species that an alkene dimer can form with a BAS:

(5) $$\Delta H{}_{r}=\Delta H{}_{d}\;+\;\Delta H{}_{s}$$

With an estimate of Δ*H*_d_=−88 kJ mol^−1^ for the heat of dimerization in the gas phase yielding a branched alkene the authors arrived at Δ*H*_s_=−285 + 88=−197 kJ mol^−1^ and assigned this enthalpy to the adsorption and formation of a C_10_ alkoxide at the BAS, see Figure 1. This assignment was based on hybrid QM:MM predictions that alkoxides are 55–57 kJ mol^−1^ more stable than the corresponding alkene π‐complexes,^[11]^ whereas later accurate QM:QM calculations showed for butenes and pentenes in H‐FER that alkoxides and π‐complexes are about equally stable.^[12]^

**Figure 1:**
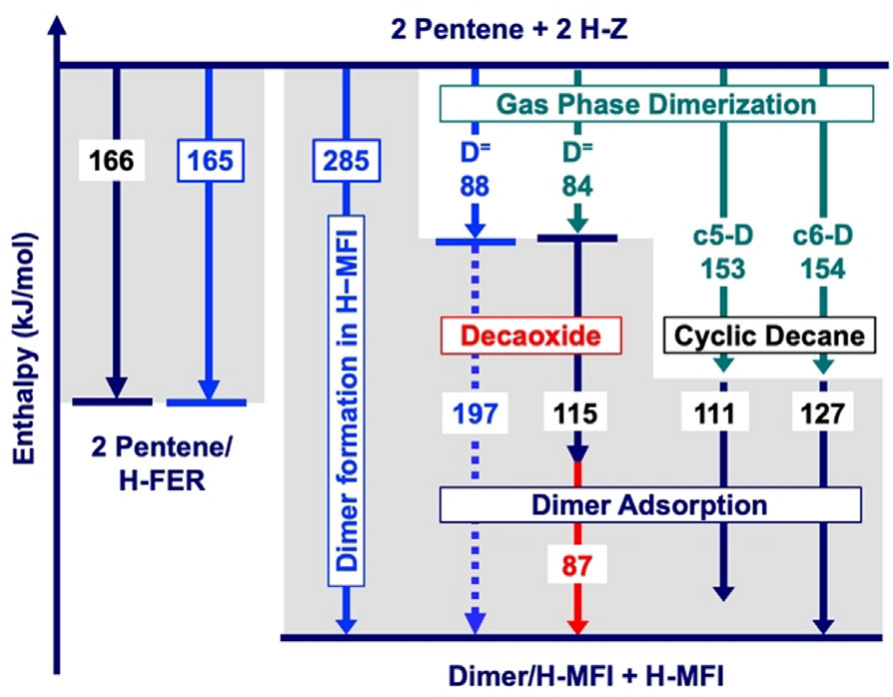
Enthalpy (323 K) decomposition for the dimerization of *trans*‐2‐pentene in H‐MFI. Blue numbers in frames are experimental results from ref. [3]. Dashed blue line is an estimate of ref. [3]. Green and black numbers are CCSD(T) and CCSD(T)‐quality QM:QM results, respectively. The red number is the discrepancy between the experimental dimer formation enthalpy and the predicted decaoxide formation enthalpy. H‐Z=Brønsted acid site, D^=^=decene, c5‐D and c6‐D=decanes featuring a cyclopentane and ‐hexane ring, respectively.

Here we propose a different assignment of the measured reaction enthalpy. We propose that dimerization of butene and pentene in acidic zeolites does not yield *branched alkenes*, as assumed in previous studies, but *cyclic alkanes*. This is based on quantum chemical gas phase heats of dimerization which are about twice as exothermic for *cyclic alkanes* than for *branched alkenes*, see Scheme 1. This is not surprising because, instead of just one, two C−C single bonds are formed from two C=C double bonds. Formation of a *cyclic alkane* instead of a *branched alkene* explains the observation of the research groups that oligomerization stops at the dimer stage[^2^, ^3^, ^4^] in a natural way—in the cyclic alkane there is no C=C double bond left.

**Scheme 1:**
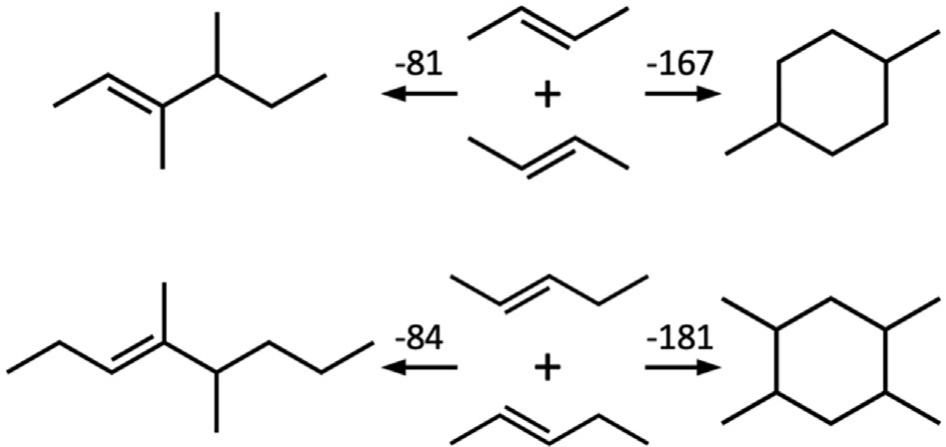
Dimerization of 2‐butene and 2‐pentene yielding either a branched alkene (left, 3,4‐dimethyl‐2‐hexene and 4,5‐dimethyl‐3‐octene, respectively) or an alkyl‐substituted cyclohexane (right, 1,4‐dimethyl‐ and 1,2,4,5‐tetramethyl‐cyclohexane, respectively). Calculated heats of dimerization at 323 K are given in kJ mol^−1^.

We have used CCSD(T)^[14]^ at 2^nd^ order Moller‐Plesset perturbation theory (MP2) structures^[15]^ for calculating dimerization enthalpies for *trans*‐2‐butene and *trans*‐2‐pentene, see Supporting Information for details. Table 1 shows the results. CCSD(T) is known to yield chemical accuracy, and comparison with experiment for the formation of propyl‐cyclopentane and ethyl‐cyclohexane from *trans*‐2‐butene as well as of pentyl‐cyclopentane and butyl‐cyclohexane from *trans*‐pentene indeed shows agreement between calculation and experiment within 2, 0, 2, and 5 kJ mol^−1^, respectively. Scheme 1 shows 3,4‐dimethyl‐2‐hexene and 4,5‐dimethyl‐3‐octene that have been suggested to form on dimerization of butene and pentene, respectively.[^2^, ^3^] Our result for the latter, −84 kJ mol^−1^, is in close agreement with the estimate of ref. [3] (−88 kJ mol^−1^). However, formation of the respective C_8_ and C_10_ alkanes which feature a cyclohexane moiety is more than twice as exothermic. There may be other alkene and alkane isomers formed, but this will not change the difference between *alkene* and *cyclic alkane* formation and, hence, will not change the conclusion. Cyclization of linear alkenes to (alkyl‐substituted) cyclopentanes and ‐hexanes has been studied by DFT as part of the hydrocarbon pool mechanism.^[16]^ Future mechanistic studies should consider a two‐step mechanism in which formation of the C_8_ or C_10_ alkene at the BAS is followed by cyclization as sketched in the Supporting Information.

**Table 1:** Heats of dimerization, Δ*H*_d_ (kJ mol^−1^), of *trans*‐2‐butene and *trans*‐2‐pentene to different products at 323 K calculated with CCSD(T) compared to experiment (in parenthesis).^[13]^

*Trans*‐2‐butene	−Δ*H*_d_ [kJ mol^−1^]	*Trans*‐2‐pentene	−Δ*H*_d_ [kJ mol^−1^]
propyl‐cyclopentane	124 (126)	pentyl‐cyclopentane	127 (125)
ethyl‐cyclohexane	151 (151)	butyl‐cyclohexane	154 (149)
3,4‐dimethyl‐3‐hexene	57	4,5‐dimethyl‐4‐octene	61
3,4‐dimethyl‐2‐hexene	81	4,5‐dimethyl‐3‐octene	84
1‐ethyl‐2‐methyl‐cyclopentane	133	1,3‐diethyl‐2‐methyl‐cyclopentane	141
1,2,3‐trimethyl‐cyclopentane	148	1‐ethyl‐2,4,5‐trimethyl‐cyclopentane	153
1,4‐dimethyl‐cyclohexane	167	1‐ethyl‐2,4‐dimethyl‐cyclohexane	170
		1,2,4,5‐tetramethyl‐cyclohexane	181

For different possible dimer species, Figure 1 compares the measured reaction enthalpy for formation of surface dimers at BAS of H‐MFI,^[3]^ Δ*H*_r_=−285 kJ mol^−1^, with the sum of our CCSD(T) gas phase dimerization enthalpies, Δ*H*_d_, and enthalpies for adsorption of the formed dimer, Δ*H*_s_, see also Table 2.

**Table 2:** CCSD(T) enthalpies of dimerization, Δ*H*_d_, MP2+ΔCC adsorption enthalpies, Δ*H*_s_, and resulting [Eq. (5)] enthalpies of formation of surface dimers, Δ*H*_r_, for different C10 alkene dimer species. The deviation, Δ, of calculated values from experiment (−285 kJ mol^−1^) is also given. All at 323 K and in kJ mol^−1^.

	−Δ*H*_d_ kJ mol^−1^	−Δ*H*_s_ kJ mol^−1^	−Δ*H*_r_ kJ mol^−1^	Δ kJ mol^−1^
**Branched alkene**: 4,5‐dimethyl‐3‐octene				
carbenium ion	84	95	179	106
alkoxide	84	115	198	87
π‐complex	84	143	227	58
**Cyclic alkanes**:				
pentyl‐cyclopentane	127	116	242	43
1‐ethyl‐2,4,5‐trimethyl‐cyclopentane	153	111	264	21
butyl‐cyclohexane	154	127	281	4
1,2,4,5‐tetramethyl‐cyclohexane	181	76	257	28

The latter are obtained with a hybrid QM:QM method^[17]^ which yields results of CCSD(T)‐quality for extended systems. It combines DFT^[18]^ including dispersion^[19]^ for the full periodic zeolite structure with MP2 for the adsorption/reaction site represented by a cluster model. For smaller cluster models of affordable size CCSD(T) calculations^[14]^ are performed, see Supporting Information for details. Energies obtained this way are termed “MP2+ΔCC”,^[12]^ their estimated uncertainty is ±4 kJ mol^−1^.^[20]^ The distribution of BAS over the different crystallographic positions of the MFI framework is not known and varies with the synthesis conditions.^[21]^ Calculations for proton exchange barriers for BAS at different T sites yield an estimated uncertainty of ±4 kJ mol^−1^ due to surface heterogeneity.[^20^, ^22^] Hence, the combined uncertainty is ±8 kJ mol^−1^. Our MP2+ΔCC calculations are performed for a BAS with Al in position T12 which is an easily accessible site at the channel intersection. Previous calculations for this site have reproduced experimental barriers for the methylation of alkenes in H‐MFI within ±4 kJ mol^−1^.^[23]^

For the first step of the surface dimerization (Figure 1), adsorption of two *trans*‐2‐pentene molecules on BASs of H‐FER [π‐complex formation, Eq. (1a)], MP2+ΔCC results (−166 kJ mol^−1^)^[12]^ and experiment (−165 kJ mol^−1^)^[3]^ are in very close agreement, thus confirming the accuracy of the hybrid QM:QM method.

The assumption that 4,5‐Dimethyl‐3‐octene is formed^[3]^ implies an adsorption enthalpy of −201 kJ mol^−1^. Our MP2+ΔCC results show that this can neither be explained by formation of a C_10_ alkoxide as assumed in refs. [3, 4]. (Δ*H*_s_=−115 kJ mol^−1^) nor with the formation of a π‐complex (−143 kJ mol^−1^) as assumed by Kondo et al.^[2]^ The deviations of the predicted enthalpies of formation of the surface dimer from the experimental reaction enthalpy, −87 and −58 kJ mol^−1^, respectively, are much larger than the estimated uncertainty of our Δ*H*_r_ calculations of ±11 kJ mol^−1^ (±3 for CCSD(T) gas phase dimerization energies, see Table 1, and ±8 kJ mol^−1^ for MP2+ΔCC adsorption energies and surface heterogeneity).

In contrast, for formation of butyl‐cyclohexane we predict an adsorption enthalpy Δ*H*_s_=−127 kJ mol^−1^, very close to the experimental result for n‐decane of −125 kJ mol^−1^,^[24]^ which yields a reaction enthalpy of Δ*H*_r_=−281 kJ mol^−1^, only 4 kJ mol^−1^ away from the measured −285 kJ mol^−1^. Formation of 1‐ethyl‐2,4,5‐trimethyl‐cyclopentane seems also possible—the predicted deviation is Δ=21 kJ mol^−1^.

We conclude from our enthalpy calculations for the formation of surface dimers that an alkyl‐substituted cyclohexane (or, less likely, cyclopentane) attached to a BAS is formed.

The formation of a *cyclic alkane* instead of a *branched alkene* explains also in a natural way the observed changes of IR spectra for the dimerization of butene[^2^, ^4^] and pentene^[3]^ in H‐MFI. Figure 2 shows the simulated spectral change for the conversion of the π‐complex of an alkene monomer (initial) into the complex of a dimer alkane with the OH group of the BAS (final) in H‐MFI. Line shapes (full width at half maximum) are taken from experiment[^2^, ^3^] and wavenumbers and intensities are obtained with DFT for the unloaded zeolite and adsorption complexes of pentene and pentane with the BAS, see Supporting Information for details. We consider pentane as representative of alkanes in general, also for dimerization products such as alkyl‐substituted cyclohexanes discussed above.

**Figure 2:**
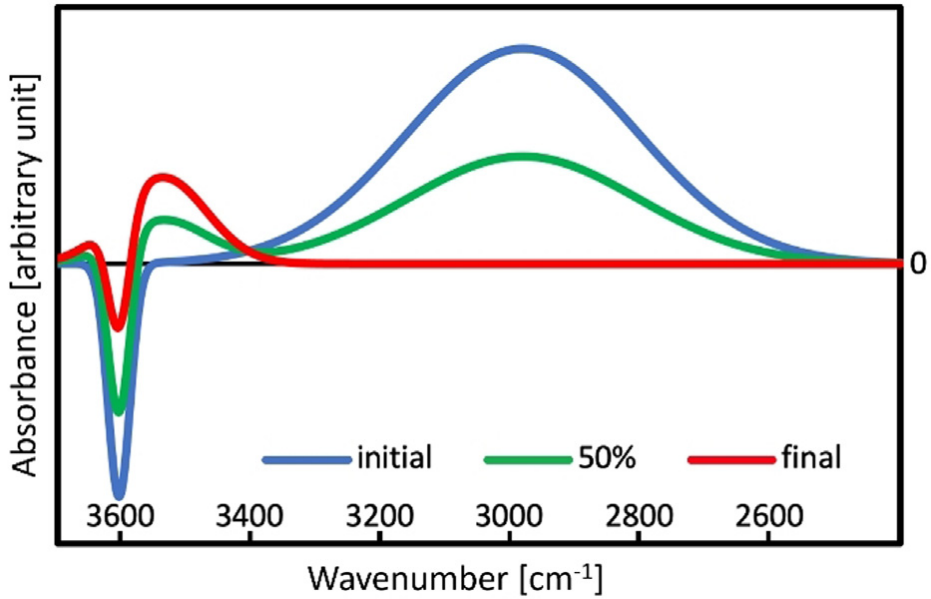
Simulated IR difference spectra for the initial, 50 % conversion, and final state of the dimerization of *trans*‐*2*‐pentene to a (cyclic) alkane in H‐MFI, based on experimental[^2^, ^3^] full width at half maximum (FWHM) values and DFT (B3LYP+D3)[^19a^, ^25^] for wavenumbers and intensities.

The broad red‐shifted band around 3000 cm^−1^ for the H‐bond in the π‐complex of the BAS OH group with the C=C double bond disappears because in the dimerization products, alkyl‐substituted cyclohexanes, there is no C=C double bond left, and a less red‐shifted OH band, typical of the interaction of the BAS OH group with alkanes,^[10]^ appears at 3450–3500 cm^−1^.

In conclusion, we have shown that, at room temperature, dimerization of butene and pentene yields C_10_ *alkanes* that feature a cycle, most likely a hexane ring, rather than linear *alkenes* as previously proposed. This finding will be crucial for rationalizing alkene oligomerization selectivity in zeolite catalysis at higher temperatures, for example, the variation of dimer selectivity over different zeolite frameworks observed for pentene in liquid phase at 473 K.^[1b]^ At these higher temperatures additional reaction steps such as β‐scission may occur.^[1]^ In future experiments, identification of cyclic dimerization products by ^13^C‐NMR should be considered, or by GC‐MS after dissolution of used catalyst samples in diluted HF.^[9b]^

## Conflict of interest

The authors declare no conflict of interest.

## Supporting information

As a service to our authors and readers, this journal provides supporting information supplied by the authors. Such materials are peer reviewed and may be re‐organized for online delivery, but are not copy‐edited or typeset. Technical support issues arising from supporting information (other than missing files) should be addressed to the authors.

Supplementary
