# Supplementary material for: Dimerization of Linear Butenes and Pentenes in an Acidic Zeolite (H‐MFI)
Source: Angew Chem Int Ed Engl. 2020 Dec 14;60(7):3529–33. doi: 10.1002/anie.202013671 (PMC7898720; doi:10.1002/anie.202013671)
Supplement: Supplementary file 1 — Supplementary [file ANIE-60-3529-s001.pdf]

Angewandte Chemie
Eine Zeitschrift der Gesellschaft Deutscher Chemiker

Supporting Information

Dimerization of Linear Butenes and Pentenes in an Acidic Zeolite (H-MFI)

*Fabian Berger and Joachim Sauer**

anie_202013671_sm_miscellaneous_information.pdf

S1. CCSD(T) calculations of dimerization energies and enthalpies

Structures are optimized with (i) density functional theory (DFT) using the PBE functional[1] augmented with the Grimme's D2 dispersion term,[2] and (ii) with second order Møller-Plesset perturbation theory (MP2) using the resolution of identity (RI) approximation,[3] all as implemented in TURBOMOLE 7.3.[4] DFT optimizations employ a triple-ζ plus polarization basis set (def2-TZVPP),[5] whereas MP2 optimizations use the cc-pVTZ basis set.[6] The domain based local pair natural orbital (DLPNO) CCSD(T) method,[7] as implemented in OCRA 4.01,[8] in combination with a two-point basis set extrapolation scheme[9] with cc-pVXZ basis sets, X=T,Q,[6] implemented in MonaLisa,[10] is used to obtain electronic energies based on MP2 optimized structures. Zero-point vibrational energies and thermal contributions to enthalpies are calculated from harmonic vibrational energies evaluated with PBE+D2.

Table S1.1. CCSD(T) energies at MP2 equilibrium structures, ΔE, zero-point vibrational energies,[a] ΔE_{ZPV}, and CCSD(T) enthalpies at 323 K,[a] ΔH_{323}, for dimerization of *trans*-2-butene to different products in kJ mol^{-1}.

Product	$-\Delta H$, Experiment[b]	$-\Delta H_{323}$	$-(\Delta E+\Delta E_{ZPV})$	$-\Delta E$
1,4-Dimethyl-cyclohexane		166.6	157.8	183.9
Ethyl-cyclohexane	151	150.5	141.2	169.7
1,2,3-Trimethyl-cyclopentane		147.9	140.7	163.8
1-Ethyl-2-methyl-cyclopentane		133.4	125.8	151.1
Propyl-cyclopentane	126	123.5	115.9	141.9
2,3-Dimethyl-2-hexene		84.7	81.9	96.3
2-Methyl-2-heptene		83.6	80.2	95.9
3,4-Dimethyl-2-hexene		81.4	78.3	93.5
2-Octene		75.2	71.7	88.4
1-Octene	61.3	65.5	61.8	79.3
3,4-Dimethyl-3-hexene		57.4	54.3	68.3

[a] Vibrational energies calculated with PBE+D2; [b] ref. [11]

Table S1.2. CCSD(T) energies at MP2 equilibrium structures, ΔE, zero-point vibrational energies,[a] ΔE_{ZPV}, and CCSD(T) enthalpies at 323 K,[a] ΔH_{323}, for dimerization of *trans*-2-pentene to different products in kJ mol^{-1}.

Product	$-\Delta H$, Experiment[b]	$-\Delta H_{323}$	$-(\Delta E+\Delta E_{ZPV})$	$-\Delta E$
1,2,4,5-Tetramethyl-cyclohexane		180.5	172.8	195.1
1-Ethyl-2,4-dimethyl-cyclohexane		169.7	161.3	186.2
1,2,3,4,5-Pentamethyl-cyclopentan		160.6	154.0	174.4
Butyl-cyclohexane	149.2	153.9	144.9	172.5
1-Ethyl-2,4,5-trimethyl-cyclopentane		153.2	146.7	167.9
1,3-Diethyl-2-methyl-cyclopentane		141.3	133.9	157.7
Pentyl-cyclopentane	124.9	126.7	119.3	144.8
2,3-Dimethyl-2-octene		87.9	85.5	98.9
2-Methyl-2-nonene		86.5	83.4	98.4
4,5-Dimethyl-3-octene		83.5	80.8	95.3
2-Decene		76.3	73.3	88.9
1-Decene	60.6	66.5	63.2	79.7
4,5-Dimethyl-4-octene		61.4	58.9	71.6

[a] Vibrational energies calculated with PBE+D2; [b] ref. [11]

S2. Hybrid QM:QM calculations for different C_{10} alkene and alkane species in H-MFI

The *hybrid MP2:(PBE+D2)+Δ_{CC}* and *MP2:(PBE+D2)* implementation applied uses a two-point basis set extrapolation scheme[9] with cc-pVXZ basis sets, X=T,Q,[6] and counterpoise corrections for the CCSD(T) and MP2 wavefunction methods[8, 12] as high-level method on cluster models and PBE+D2[1-2] as low-level method for the full periodic structures, see ref.[13] for details. DFT with periodic boundary conditions was applied using the VASP code, version 5.3.5.[14] This code uses plane-wave basis sets to describe valence electrons and the projector-augmented wave (PAW) method[15] to include the effects of core electrons. As previously,[13, 16] a kinetic energy cutoff of 400 eV and Γ-point sampling were used together with the PBE exchange-correlation functional, and D2 dispersion correction.[2, 17] The SCF energy convergence criterion was 10^{-7} eV, and structure optimizations were considered converged when the maximum force acting on the atoms was less than 10^{-3} eV Å^{-1}.

The hybrid energy is defined as

$$\Delta E(\mathrm{S}) = \Delta E_{\mathrm{HL}}(\mathrm{C}_{\mathrm{MP2}}) - \Delta E_{\mathrm{LL}}(\mathrm{C}_{\mathrm{MP2}}) + \Delta E_{\mathrm{LL}}(\mathrm{S})\ (+\Delta_{\mathrm{CC}}) \quad (1)$$

with an optional coupled cluster correction

$$\Delta_{\mathrm{CC}} = \Delta E_{\mathrm{CCSD(T)}}(\mathrm{C}_{\mathrm{CC}}) - \Delta E_{\mathrm{MP2}}(\mathrm{C}_{\mathrm{CC}}). \quad (2)$$

Table S2. MP2+Δ_{CC} enthalpies for adsorption of dimerization products in H-MFI at 323 K and 0K,[a] ΔH_{323} and ΔH_0 , respectively, hybrid MP2:PBE+D2 and PBE+D2 energies as well as coupled cluster corrections, Δ_{CC}, all in kJ/mol.

			−ΔE		
	$-\Delta H_{323}$[a]	$-\Delta H_0$[a]	Δ_{CC}	MP2:PBE+D2	PBE+D2
**Branched alkene:** 4,5-dimethyl-3-octene					
carbenium ion	94.7	93.8	–5.3	94.3	146.1
alkoxide	115.4	110.9	–0.6	131.1	156.6
π-complex	143.3	144.0	6.2	151.3	165.6
**Cyclic alkanes**					
Pentyl-cyclopentane	115.6	117.8	(–0.1)[b]	121.8	140.1
1-Ethyl-2,4,5-trimethyl-cyclopentane	110.9	111.5	(–0.1)[b]	118.7	127.4
Butyl-cyclohexane	127.4	130.3	(–0.1)[b]	133.3	144.0
1,2,4,5-Tetramethyl-cyclohexane	76.4	78.6	(–0.1)[b]	83.4	102.6

[a] Vibrational energies calculated with PBE+D2, [b] Δ_{CC} corrections for adsorption for propane, n-butane and i-butane in H-MFI are as small as -0.1 kJ/mol, ref.[13] and ignored in the present ΔH_{323} results.

S3. Extrapolated heats of adsorption for C_8 and C_{10} alkanes and alkenes interacting with H-MFI or H-FER

For an estimate of the heat of adsorption of C_{10} alkanes we use experimental information only, see Table S3. We add 5 x (−12±2) kJ/mol to the measured values[18] for *i*-pentane −64 kJ/mol) and *n*-pentane which yields estimates of −124±10 and −130±10 kJ/mol for *i*-decane and *n*-decane, respectively. The direct measurement for *n*-decane,[19] −125 kJ/mol, is within the range extrapolated values. There is also a calorimetric measurement for cyclopentane in silicalite,[20] −58 kJ/mol. Adding 5 x (−12±2 kJ/mol) for five additional CH_2 groups as well as −11±1 kJ/mol for the presence of a BAS[21] yields −129 ± 11 kJ/mol, within the range of extrapolated values for *n*- and *i*-decane.

Table S3. Estimated enthalpies for the Interaction of C_8 and C_{10} alkanes with Brønsted acid sites (BAS), ΔH_s (kJ/mol). Uncertainty values include the uncertainty connected with the increment for an additional CH_2 group,[21b] Δ_{CH2}, and for the presence of a BAS in Silicalite,[21a] ±2 and ±1 kJ/mol, respectively.

Zeolite	alkane	$-\Delta H_s$	$n(-\Delta_{CH2})$	$-\Delta H_s$ (C_8)	$n(-\Delta_{CH2})$	$-\Delta H_s$ (C_{10})
H-MFI	*i*-pentane [a]	64[a]	3 x 12	100±6	5 x 12	124±10
H-MFI	*n*-octane [b]			105 [b]	2 x 12	129±4
H-MFI	*n*-decane [b]					125 [b]
Silicalite	cyclopentane [d]	58	3 x 12	105±7 [c]	5 x 12	129±11 [c]
Silicalite	cyclohexane [d]	64	2 x 12	99±5 [c]	4 x 12	123±9 [c]

[a] Eder et al.[18]; [b] 811 K, Haag[19]; [c] Includes 11±1 kJ/mol for presence of BAS;[21] [d] Stach et al.[20].

S4. Simulation of IR spectra

A model consisting of 15 TO_4 units was adopted for H-MFI with a Al12-O8H-Si3 Brønsted site. Linking hydrogen atoms and adjacent oxygen atoms have been fixed to maintain the structure of the optimized periodic zeolite system of H-MFI.[22] For the complexes of pentane and pentene with the OH group of the BAS (Figure S1) DFT structure optimizations and harmonic wavenumber calculations were performed using PBE+D2[1-2] and B3LYP+D3[2, 23] with the (def2-)TZVPP basis set.[5]

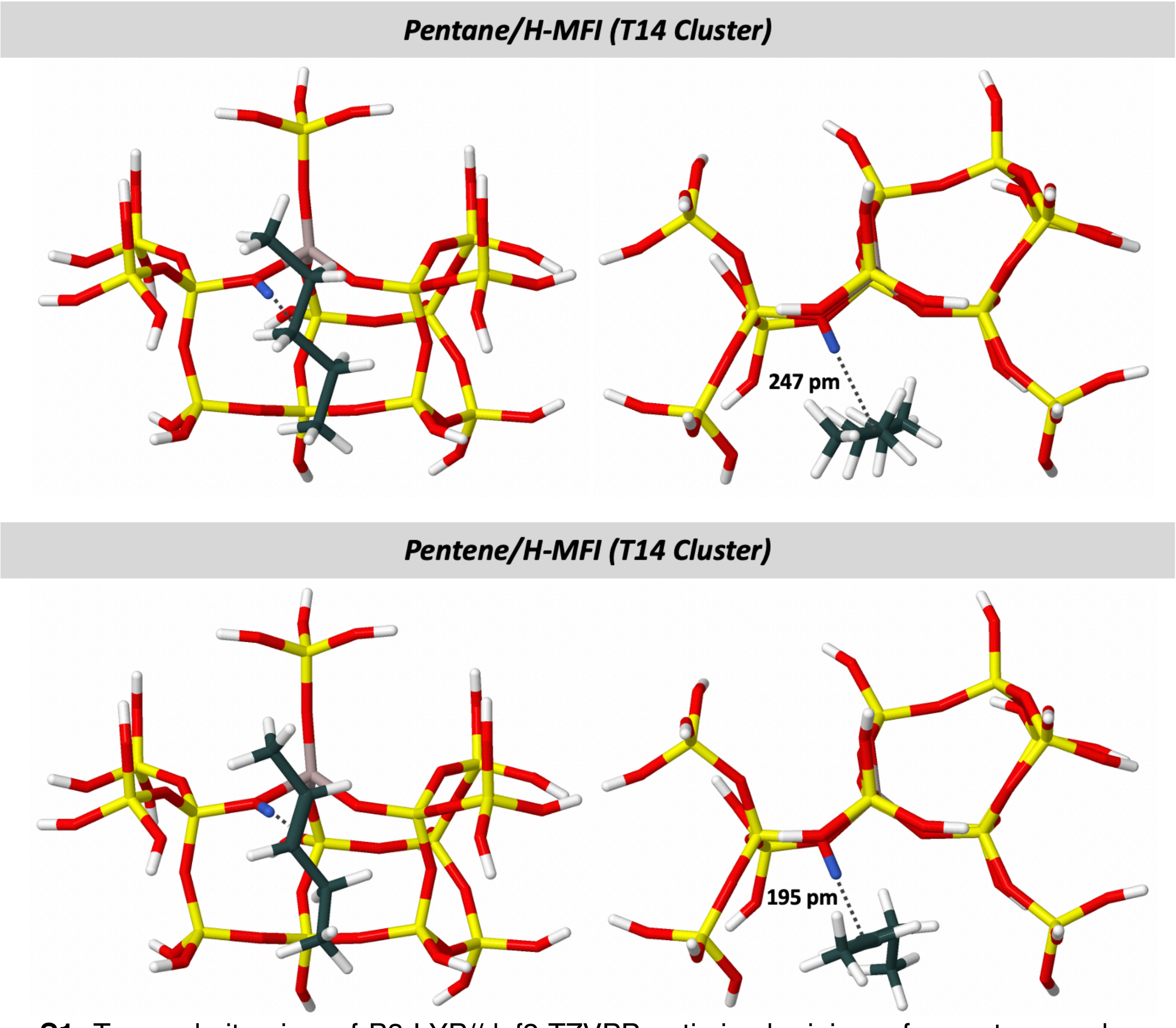

Figure S1. Top and site view of B3-LYP//def2-TZVPP optimized minima of *n*-pentane and *n*-pent-2-ene. Color code: yellow – silicon, red – oxygen, white – hydrogen, gray – aluminum, black – carbon, and blue- interacting BAS hydrogen.

For the PBE+D2[1-2] and B3LYP+D3[2, 23] calculations with (def2-)TZVPP basis sets[5] the Turbomole 7.3[4] and MonaLisa[10] codes were employed. The SCF convergence criterion was 10^{-8} E_H and the optimization threshold 10^{-6} E_H $Å^{-1}$. To account for anharmonicities and systematic errors in harmonic

force constants, harmonic wavenumbers have been scaled with factors 0.9854 and 0.9609 for PBE+D2 and B3LYP+D3, respectively. As before,[24] the factors have been obtained as the ratio of calculated harmonic wavenumbers, ω, and observed fundamentals, ν, for the average of the symmetric and antisymmetric stretching modes of H_2O according to

$$f = (\nu_s + \nu_{as})/ (\omega_s + \omega_{as}) \quad (1)$$

Table S4 shows the data used for simulation of IR spectra. From experiments, wavenumbers and line shapes (full width at half maximum) are taken, whereas (scaled) wavenumbers and intensities are taken from DFT calculations.

Table S4. IR data for alkanes and alkenes interacting with BAS in H-MFI. Vibrational wavenumbers, ν (cm^{-1}) and full width at half maximum, FWHM in cm^{-1}, from experiments as well as B3LYP+D3 and PBE+D2 results for (scaled) wavenumbers (cm^{-1}) and intensities (km/mol).

	ZOH		Alkane/ZOH			Alkene/ ZOH		
	v	FWHM		v	FWHM		v	FWHM
Spoto[a]	3609		heptane	3474	160	propene	3070	420
Kondo[b]	3610		C4-C4	3500		butene	3100	
Lercher[c]	3610	40[d]	C5-C5	3450		trans-2-pentene	3100	
		Intens.[e]			Intens.[e]			Intens.[e]
PBE+D2 (0.9854)[f]	3595	132.8 (1.00)	pentane	3478	356.5 (2.68)	pentene	2791	1542.1 (11.61)
B3LYP+D3 (0.9609)[f]	3606	132.6 (1.00)	pentane	3539	340.2 (2.56)	pentene	2983	1283.6 (9.68)

[a] Ref.[25]; [b] Ref.[26]; [c] Ref.[27]; [d] Derived from figure 2 of ref. [27]; [e] relative intensities in parenthesis; [f] scale factor.

S5. Reaction scheme for alkene dimerization

The structures in the grey shaded area are either transition structures or energetically high intermediates

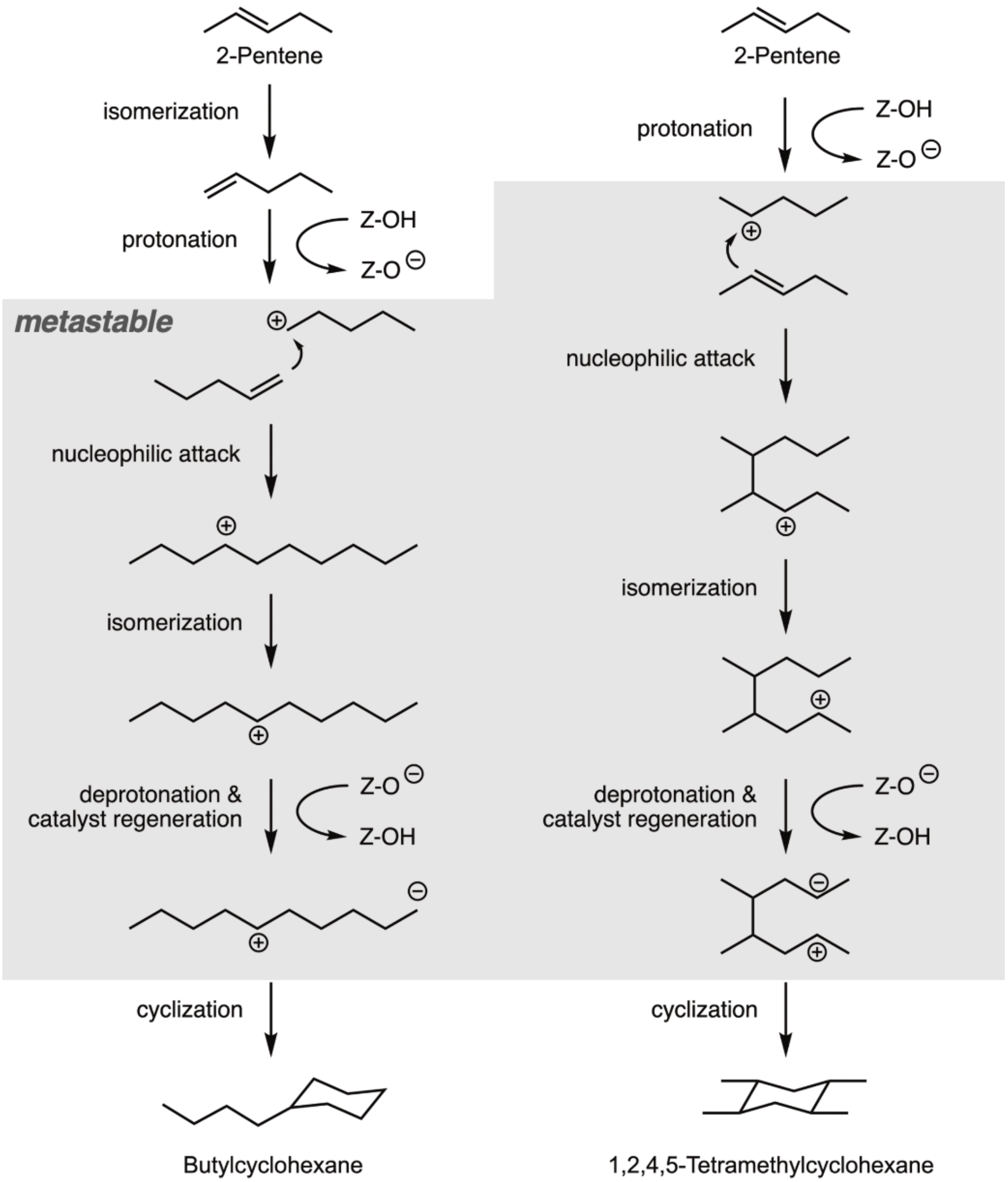

References

[1] J. P. Perdew, K. Burke, M. Ernzerhof, *Phys. Rev. Lett.* **1996**, *77*, 3865-3868.
[2] S. Grimme, *J. Comput. Chem.* **2006**, *27*, 1787-1799.
[3] C. Hättig, F. Weigend, *J. Chem. Phys.* **2000**, *113*, 5154-5161.
[4] S. G. Balasubramani, G. P. Chen, S. Coriani, M. Diedenhofen, M. S. Frank, Y. J. Franzke, F. Furche, R. Grotjahn, M. E. Harding, C. Hättig, A. Hellweg, B. Helmich-Paris, C. Holzer, U. Huniar, M. Kaupp, A. M. Khah, S. K. Khani, T. Müller, F. Mack, B. D. Nguyen, S. M. Parker, E. Perlt, D. Rappoport, K. Reiter, S. Roy, M. Rückert, G. Schmitz, M. Sierka, E. Tapavicza, D. P. Tew, C. v. Wüllen, V. K. Voora, F. Weigend, A. Wodyński, J. M. Yu, *J. Chem. Phys.* **2020**, *152*, 184107.
[5] F. Weigend, R. Ahlrichs, *Phys. Chem. Chem. Phys.* **2005**, *7*, 3297-3305.
[6] a) T. H. Dunning, *J. Chem. Phys.* **1989**, *90*, 1007-1023; b) D. E. Woon, T. H. Dunning, *J. Chem. Phys.* **1993**, *98*, 1358-1371.
[7] C. Riplinger, F. Neese, *J. Chem. Phys.* **2013**, *138*, 034106.
[8] F. Neese, *Wiley Interdiscip. Rev. Comput. Mol. Sci.* **2012**, *2*, 73-78.
[9] a) F. Jensen, *Theor. Chem. Acc.* **2005**, *113*, 267-273; b) T. Helgaker, W. Klopper, H. Koch, J. Noga, *J. Chem. Phys.* **1997**, *106*, 9639-9646.
[10] F. A. Bischoff, M. Alessio, F. Berger, M. John, M. Rybicki, J. Sauer, Humboldt-University: Berlin, www.chemie.hu-berlin.de/de/forschung/quantenchemie/monalisa/ **2019**.
[11] H. Y. Afeefy, J. F. Liebman, S. E. Stein, in *NIST Chemistry WebBook, NIST Standard Reference Database Number 69* (Eds.: P. J. Linstrom, W. G. Mallard), **2018**.
[12] P. Pinski, F. Neese, *J. Chem. Phys.* **2018**, *148*, 031101.
[13] M. Rybicki, J. Sauer, *J. Am. Chem. Soc.* **2018**, *140*, 18151-18161.
[14] G. Kresse, J. Furthmuller, *Phys. Rev. B Condens. Matter* **1996**, *54*, 11169-11186.
[15] G. Kresse, D. Joubert, *Phys. Rev. B Condens. Matter* **1999**, *59*, 1758-1775.
[16] G. Piccini, M. Alessio, J. Sauer, *Angew. Chem. Int.* **2016**, *55*, 5235-5237.
[17] T. Kerber, M. Sierka, J. Sauer, *J. Comput. Chem.* **2008**, *29*, 2088-2097.
[18] F. Eder, M. Stockenhuber, J. A. Lercher, *J. Phys. Chem. B* **1997**, *101*, 5414-5419.
[19] W. O. Haag, in *Zeolites and Related Microporous Materials: State of the Art 1994. Proceedings of the 10th International Zeolite Association Meeting, Vol. 84* (Eds.: J. Weitkamp, H. G. Karge, H. Pfeifer, W. Hölderich), Elsevier, Amsterdam, **1994**, pp. 1375-1394.
[20] H. Stach, U. Lohse, H. Thamm, W. Schirmer, *Zeolites* **1986**, *6*, 74-90.
[21] a) F. Eder, J. A. Lercher, *Zeolites* **1997**, *18*, 75-81; b) J. Sauer, in *On catalysis, Vol. 2* (Eds.: W. Reschetilowski, W. Hönle), VWB Verlag für Wissenschaft und Bildung, Leipzig, **2010**, pp. 136-161.
[22] H. Van Koningsveld, H. Van Bekkum, J. C. Jansen, *Acta Crystallogr. B* **1987**, *43*, 127-132.
[23] a) A. D. Becke, *J. Chem. Phys.* **1993**, *98*, 5648-5652; b) C. Lee, W. Yang, R. G. Parr, *Phys. Rev. B Condens. Matter* **1988**, *37*, 785-789.
[24] X. Yu, E. Emmez, Q. Pan, B. Yang, S. Pomp, W. E. Kaden, M. Sterrer, S. Shaikhutdinov, H.-J. Freund, I. Goikoetxea, R. Wlodarczyk, J. Sauer, *Phys. Chem. Chem. Phys.* **2016**, *18*, 3755-3764.
[25] G. Spoto, S. Bordiga, G. Ricchiardi, D. Scarano, A. Zecchina, E. Borello, *J. Chem. Soc. Faraday Trans.* **1994**, *90*, 2827-2835.
[26] J. N. Kondo, S. Liqun, F. Wakabayashi, K. Domen, *Catal. Letters* **1997**, *47*, 129-133.
[27] S. Schallmoser, G. L. Haller, M. Sanchez-Sanchez, J. A. Lercher, *J. Am. Chem. Soc.* **2017**, *139*, 8646-8652.
